# Aortic Compression

**Published:** 1911-01-28

**Authors:** 


### Editor's Letter-Box.

### AORTIC COMPRESSION.

*To the Editor of* The Hospital.

A correspondent writes: "In The Hospital this week mention is made of a method of constricting the abdominal aorta in cases of post-partum hæmorrhage by a rubber band known as Momberg's belt. I should like to put in a plea for the method of compression first advocated by the late Mr. G. N. G. Griffith. It is an easy and efficient way of stopping uterine hæmorrhage. The patient lies on her back, with the abdominal muscles relaxed as far as possible. The attendant compresses the aorta, just below the umbilicus and before its bifurcation, with either his finger and thumb, or the first and second fingers. By keeping the aorta firmly pressed against the spine the flow of blood can be perfectly regulated. Strangulation of the intestines cannot possibly occur, nor any other untoward result, and the patient is subjected to little or no discomfort. I have used this means of compression many times and have never known it fail."

